# Diversity of use and local knowledge of wild and cultivated plants in the Eastern Cape province, South Africa

**DOI:** 10.1186/s13002-017-0173-8

**Published:** 2017-08-08

**Authors:** Alfred Maroyi

**Affiliations:** Medicinal Plants and Economic Development (MPED) Research Center, Department of Botany, University of Fort Hare, Private Bag X1314, Alice, 5700 South Africa

**Keywords:** Eastern cape province, Plant biodiversity, Traditional ecological knowledge, Useful plants

## Abstract

**Background:**

Traditional ecological knowledge among indigenous communities plays an important role in retaining cultural identity and achieving sustainable natural resource management. Hundreds of millions of people mostly in developing countries derive a substantial part of their subsistence and income from plant resources. The aim of this study was to assess useful plant species diversity, plant use categories and local knowledge of both wild and cultivated useful species in the Eastern Cape province, South Africa.

**Methods:**

The study was conducted in six villages in the Eastern Cape province, South Africa between June 2014 and March 2017. Data on socio-economic characteristics of the participants, useful plants harvested from the wild, managed in home gardens were documented by means of questionnaires, observation and guided field walks with 138 participants.

**Results:**

A total of 125 plant species belonging to 54 genera were recorded from the study area. More than half of the species (59.2%) are from 13 families, Apiaceae, Apocynaceae, Araliaceae, Asparagaceae, Asphodelaceae, Asteraceae, Fabaceae, Lamiaceae, Malvaceae, Myrtaceae, Poaceae, Rosaceae and Solanaceae. More than a third of the useful plants (37.6%) documented in this study are exotic to South Africa. About three quarters of the documented species (74.4%) were collected from the wild, while 20.8% were cultivated and 4.8% were spontaneous. Majority of the species (62.4%) were used as herbal medicines, followed by food plants (30.4%), ethnoveterinary medicine (18.4%), construction timber and thatching (11.2%). Other minor plant use categories (1–5%) included firewood, browse, live fence, ornamentals, brooms and crafts.

**Conclusion:**

This study demonstrated that local people in the Eastern Cape province harbour important information on local vegetation that provides people with food, fuel and medicines, as well as materials for construction and the manufacturing of crafts and many other products. This study also demonstrated the dynamism of traditional ecological knowledge, practices and beliefs of local people demonstrated by the incorporation of exotic plants in their diets and indigenous pharmacopoeia.

## Background

Plant biodiversity provide humans with four categories of ecosystem goods and services which are provisioning, regulating, supporting and cultural services [1]. Their direct provisioning services to humans are food, fodder, medicines, timber, fuelwood and grazing, while regulating services include moderating air and water quality and erosion control [2]. Plant species also play a vital role in supporting services such as soil formation, and nutrient and water cycling and in cultural services, including traditional human knowledge systems [2]. Therefore, plant biodiversity is required to fulfill various human daily livelihood needs. According to Uprety et al. [3] hundreds of millions of people, mostly in developing countries, derive a substantial part of their subsistence and income from wild plant products. Research by Sunderland [4] revealed that plant biodiversity provides an important safety net during times of food insecurity, particularly during times of low agricultural production, during other seasonal or cyclical food gaps or during periods of climate induced vulnerability. Plant resources collected from the wild are an important safety net and source of livelihood needs especially for the poor and those people who live in marginalized areas who rely on them for food, fuelwood, medicines and building materials. In sub-Saharan Africa, the loss of traditional ecological knowledge (TEK) among indigenous communities has been described as one of the greatest challenges to the continent for retaining cultural continuity and achieving sustainable natural resource management [5–11]. Berkes et al. [12] defined TEK as a cumulative body of knowledge, practice and belief, evolving by adaptive processes and handed down over generations by cultural transmission, about the relationships between living beings and their environment. Similarly, research by Harisha et al. [13] revealed that TEK is a key element of the social capital to produce food, primary healthcare and in shaping local visions and perceptions of the surrounding environment and society. Traditional ecological knowledge has contributed to conservation of biodiversity, rare species and protected areas as well as to sustainable natural resource use in several countries throughout the world [14].

Given the widespread decline of TEK about plant diversity in sub-Saharan Africa [6, 7, 9, 14], there is therefore, need to document this traditional knowledge which has accumulated over centuries and transferred orally from generation to generation. Van Wyk and Gericke [15] argued that changes in the socio-cultural and environmental landscapes over the past decades resulted in the erosion of TEK of local communities. Such changes include improved access to modern health care services, improved education system, shifts of populations from rural to urban centres, changes from subsistence farming to cash-crop production, reliance on migrant labour and unprecedented environmental degradation. Research by Van Wyk and Gericke [15] revealed that the use of plants by local people is still a relatively underdeveloped discipline in southern Africa and knowledge of indigenous plant use in the region needs urgent scientific documentation before it is irretrievably lost to future generations. It is within this context that assessment of plant diversity, use and local knowledge of both wild and cultivated species was carried out in the Eastern Cape province, South Africa.

The Eastern Cape province is approximately 170,000 km^2^ in area and is inhabited by about 6.7 million people, which equals about 13.8% of both the total population and the total land area of South Africa [16]. This province is predominantly inhabited by isiXhosa speaking people of Cape Nguni descent. The Eastern Cape province includes two of the former homeland areas, namely Ciskei and Transkei out of the 13 former racially-defined homelands or Bantustan areas of South Africa. One of the Apartheid government’s acts of segregation was the Bantu Authorities Act of 1951, which legalized the deportation of Black people into designated homelands. Black people were forcibly removed from urban areas and white farms to those areas demarcated as homelands. The Ciskei and Transkei are today characterized by landlessness, pervasive chronic poverty, low levels of education, economic activity, vulnerability, lack of basic services, a dearth of employment opportunities and high levels of dependency on welfare [17]. An estimated 72% of the population in the Eastern Cape province live below the poverty line, which is more than the national average of 60% and this is attributed to the legacies of Apartheid; where the Eastern Cape provincial administration inherited the largely impoverished and corrupt former Ciskei and Transkei homelands [18]. Research by Westaway [17] revealed that the majority of households in the Eastern Cape province spend most of their income on food and there is clear evidence of growing food insecurity as measured by the number of meals consumed and the quantity and variety of foods eaten. Most people in the province live in rural areas, the contribution of agriculture to local livelihoods is low in the entire province and has been in decline for several decades [19]. However, research by Shackleton et al. [20] revealed that local people’s livelihoods in the province are centred on grasslands and forests for fodder, wild foods, firewood, medicinal plants and fibre species for weaving. Therefore, the current study was undertaken to assess diversity of use and local knowledge of wild and cultivated plants in the Eastern Cape province. This study was carried out to gather support for the hypothesis that the Eastern Cape province is historically linked to peasant and indigenous people who derive their livelihoods from the surrounding plant resources contributing to TEK about plant biodiversity in the province. Other researchers, for example, Harisha et al. [13] and Irakiza et al. [14] argued that TEK is an expression of cultural diversity which has been instrumental in the characterization of plant biodiversity, promotion of resilience of ecosystems, conservation and management of natural resources and meeting basic livelihood needs of local communities. Therefore, insight on useful plant species diversity, plant use categories and TEK associated with such plant species in the Eastern Cape province was documented in this study.

## Methods

### Study area

**Fig. 1:**
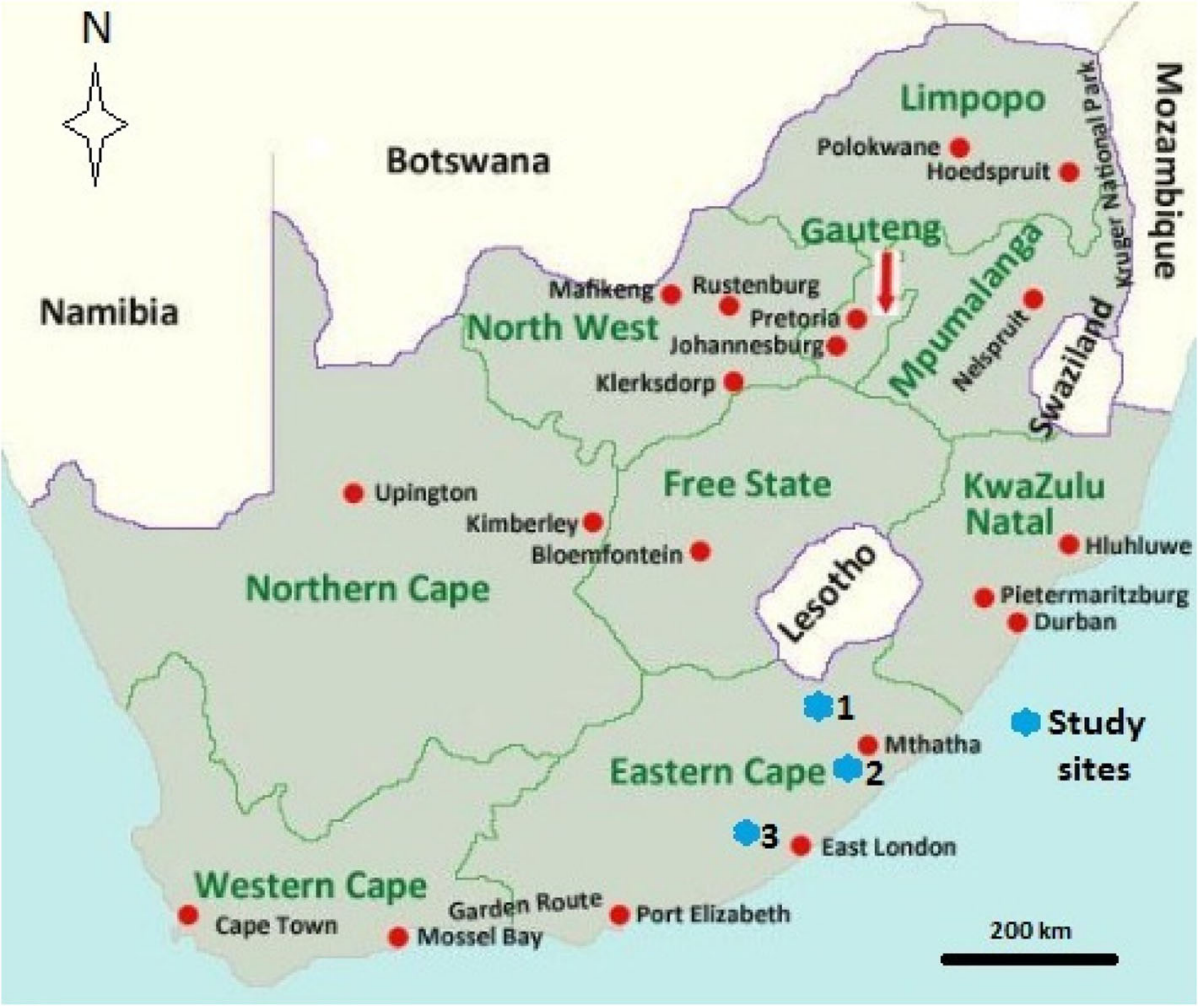
Map of South Africa illustrating the geographical position of the study areas

The study was conducted in six villages: Mpetsheni and Ngxoto villages (study site 1, see Fig. 1) in the Elundi Local Municipality; Colosa and Mangathi villages, study site 2 in the Mbhashe Local Municipality and Ngqele and KwaKhayalethu villages (study site 3, Fig. 1) in the Raymond Mhlaba Local Municipality. Study sites 1 and 2 are situated in the former Transkei homeland while study site 3 is situated in the former Ciskei homeland. Study sites 1 and 2 are predominantly rural with the dominant land use practise being rearing of livestock and dryland crop production. Major crops cultivated in the study area include maize (*Zea mays* L.), potatoes (*Solanum tuberosum* L.), cabbage (*Brassica oleracea* L.), spinach (*Spinacia oleracea* L.), beetroot (*Beta vulgaris* L.) and carrots (*Daucas carota* L.). The majority of the inhabitants (at least 87%) in the study sites are traditional isiXhosa speaking people who are highly dependent on natural resources for their livelihoods [21]. According to Chalmers and Fabricus [22], the vegetation of Mbhashe Local Municipality can best be described as grassland-woodland-forest mosaic, with a clear distinction between the boundaries of forests, woodland and grassland because of the effects of fire and clearing for cultivation. Mucina and Rutherford [23] described the vegetation of Ugie and Maclear, the two towns nearest to study site 1 as Drakensberg foothill moist grassland. This vegetation type occurs at an altitude of 880–1860 m above sea level with the landscape characterized by moderately rolling hills [23]. The grassland of both study sites 1 and 2 generally occurs on the high ridges, whereas the forest patches occur on the moist deeper soils in the protected valleys with the woodland in transition zone between the forest and the grassland. Study sites 1 and 2 are located in a climatic transition zone between the temperate south coast and the subtropical north coast of South Africa with average annual rainfall of 1069 mm, average winter and summer temperatures of 21.5 °C and 24 °C respectively [24].

The climate of study site 3 in the Raymond Mhlaba Local Municipality can be described as mild with unevenly distributed annual rainfall within the area with most rain falling during summer months from October to March. Annual rainfall ranges from 500 mm to 1000 mm, with mountainous areas receiving the highest rainfall and low to medium areas characterized by low to average annual rainfall [25, 26]. The climate varies from hot in summer to extreme cold in winter with heavy frost and snowfall along the hilly areas. The temperature ranges from of 4 °C in July to 38 °C in February [25, 26]. Raymond Mhlaba Local Municipality is characterized by a variety of land uses ranging from commercially oriented rangeland stock farming to small-scale vegetable and crop production and stock farming [25]. Other economic activities in the Raymond Mhlaba Local Municipality include tourism, forestry and sheep and wool production [26]. According to the vegetation classification of Mucina and Rutherford [23], the Raymond Mhlaba Local Municipality has grassland, succulent thicket and *Acacia* thornveld dominated by *Acacia karroo* Hayne*, Aloe ferox* Mill.*, Aloe aborescens* Mill.*, Diospyros dichrophylla* (Grand.) De Winter*, Eragrostis curvula* (Schrad.) Nees, Euphorbia spp., *Melinis nerviglumis* (Franch.) Zizka and *Olea europaea* L. ssp. *africana* (Mill.) P. S. Green.

### Data collection

Data on diversity of use and local knowledge of wild and cultivated plants in six villages namely Colosa, KwaKhayalethu, Mangathi, Mpetsheni, Ngqele and Ngxoto in the former Ciskei and Transkei homelands (Fig. 1) were collected by means of questionnaires between June 2014 and March 2017. Participatory rural appraisal (PRA) methods were used [27, 28] aimed at incorporating the TEK and opinions of community members about useful plant species diversity and plant use categories species in the Eastern Cape province. Interviews and dialogue with the participants were an integral part of this research, enabling the researcher to understand a lot on the people’s resource use culture [27, 28]. The PRA exercises, though they are difficult to quantify, provide a valuable insight into the multiple meanings, dimensions and experiences of local people with plant resources around them. This research technique captures information that standard plant use assessment methods are likely to miss. Open-ended methods, such as unstructured interviews and discussion groups allow the emergence of issues and dimensions that are important to the community but not necessarily known to the researcher, thus allowing unanticipated themes to be explored by the interviewer [29].

One hundred and thirty eight (Table 1) randomly selected participants who took part in this study were requested to sign University of Fort Hare (MAR011) informed consent form and researchers also adhered to the ethical guidelines of the International Society of Ethnobiology (www.ethnobiology.net). The majority of these participants (65.9%) were females and their age range was from 19 to 81 years (Table 1). More than 80% of the participants live below the national poverty line and 62.3% of the households had total income of less than R1000.00 (US$87.00) per month (Table 1). Close to three quarters of the participants (73.9%) were unemployed, with 63.0% surviving on social grants (Table 1).

**Table 1:** Socio-economic characteristics of the study sample, *N* = 138

Socio-economic variable	Value
Gender: Female	65.9%
Male	34.1%
Age	19–81 years (median 57 years)
People living in poverty	80.4%
Household income (<R1000.00 (US$87.00)	62.3%
Unemployed	73.9%
Dependent on social grants	63.0%
Household size	1–12 people (average 4.5)

The aim and objectives of the study were presented to the participants before being interviewed. Results obtained via use of the questionnaires were complemented with personal observation, informal discussions and guided field walks or surveys with the participants. Interview discussions took place in the local language, isiXhosa and were translated into English with the help of an interpreter. During the interviews we documented information on names of useful plants, including species grown and managed in home gardens, uses, plant parts used and preparation of useful plants. Plant species were identified in the field and the taxon names conform to those of Germishuizen et al. [30]. Unknown plant species were collected, pressed, oven-dried and identified by taxonomists at the Giffen Herbarium (UFH) at the University of Fort Hare and Schonland Herbarium (GRA) at Rhodes University, Grahamstown, South Africa.

### Data management and analysis

The data collected were entered in Microsoft Excel 2007 file and this data were used to determine frequencies and other descriptive statistical patterns. Box plots featuring medians, first and third quartiles and a range of plant use categories were computed using Palaeontological Statistics [31], version 3.06. The majority of the data collected in this study were descriptive and qualitative in nature, and therefore, were explained directly. Interview responses from participants were coded and sorted into themes, paying particular attention to inconsistencies and unique statements.

## Results and discussion

### Plant diversity

**Fig. 2:**
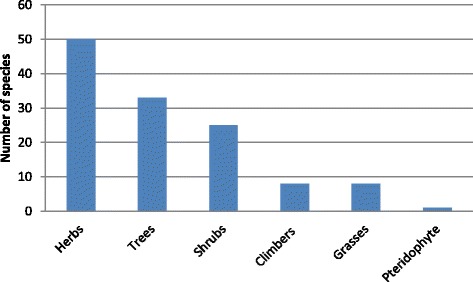
Growth forms observed in the Eastern Cape province

A total of 125 plant species were recorded in the Eastern Cape province (Table 2), with herbs, trees and shrubs having the most species (Fig. 2). Pteridophytes and gymnosperms were represented by a single species each, that is, *Cheilanthes hirta* (family Pteridaceae) and *Podocarpus latifolius* (family Podocarpaceae) respectively. A large number (59.2%, *n* = 74) of the plant species recorded in this study are from 13 families (Table 3). The other 41 families had less representation, between one and two species each. Plant families with the highest number of species were: Asteraceae (10 species), Fabaceae and Solanaceae (nine species each), Poaceae (eight species), Asphodelaceae (seven species), Rosaceae (five species), Apiaceae, Apocynaceae, Asparagaceae, Lamiaceae and Myrtaceae (four species each), Araliaceae and Malvaceae (three species each) (Table 3). All these plant families with the exception of Araliaceae are among the largest plant families in South Africa, characterized by more than 100 species each [30]. Of the 125 species identified in this study (Table 2), 93 species (74.4%) were collected from the wild, while 26 species (20.8%) were cultivated and six species (4.8%) were spontaneous. More than a third of the plant species (37.6%) documented in this study are exotic to South Africa, while the remainder (62.4%) are native plants.

**Table 2:** Useful plant species recorded in in the Eastern Cape province, South Africa

Species name and voucher number	Family	Vernacular name (isiXhosa)	Growth form	Wild/cultivated	Frequency (%)^b^	Main use	Parts used	Cured diseases	Other uses
^a^ *Acacia baileyana* F. Muell.; AM 1421	Fabaceae	Iwatlisi	Tree	W	2.9	T	Stems		
Acacia caffra (Thunb.) Wild.; AM 1504	Fabaceae	Umthole	Tree	W	3.6	T	Stems		
^a^Acacia dealbata Link.; AM 1422	Fabaceae	Idywabasi	Tree	W	4.3	T	Stems		Fi
*Acacia karroo* Hayne; AM 1423	Fabaceae	Umnga	Tree	W	21.7	Fi	Bark, leaves, stems	Boils, diarrhoea, haemorrhage, ringworm, thrush, tuberculosis (TB)	B, EVM, M
^a^Acacia mearnsii De Wild.; AM 1424	Fabaceae	Idywabasi	Tree	W	7.2	T	Bark, leaves, stems	Diarrhoea	Fi, M
Acokanthera oblongifolia (Hochst.) Codd; AM 1451	Apocynaceae	Inxinebe	Tree	W	8.7	M	Leaves	Headache, snakebite	EVM
*Agapanthus africanus* Hoffmanns; AM 1432	Alliaceae	Isicakathi	Herb	W	5.1	M	Leaves	Antiseptic, rash, stomach problems	
^a^ *Agave americana* L.; AM 1452	Asparagaceae	Ikhamanga	Shrub	WC	5.8	O	Leaf sap, whole plant	High blood pressure	M
Alepidea amatymbica Eckl. & Zeyh.; AM 1494	Apiaceae	Iqwili	Herb	W	25.4	M	Roots	Abdominal pains, fever, pimples, wounds	
Alepidea serrata Eckl. & Zeyh.; AM 1505	Apiaceae	Ubulawa	Herb	W	1.4	M	Roots	Cough	
^a^ *Allium cepa* L.; AM 1507	Alliaceae	Itswele	Herb	C	40.6	F	Bulb		
^a^ *Allium sativum* L.; AM 1508	Alliaceae	Ivimbampunzi	Herb	C	11.6	F	Bulb	Cough	M
*Aloe arborescens* Mill.; AM 1493	Asphodelaceae	Ingcelwane	Shrub	W	12.3	M	Leaf gel	Constipation, dry skin, wounds	EVM
Aloe ciliaris Haw.; AM 1506	Asphodelaceae	Intelezi	Shrub	W	1.4	M	Leaf gel	Wounds	
*Aloe ferox* Mill.; AM 1409	Asphodelaceae	iKhala	Shrub	W	38.4	M	Leaf gel	Boils, dry skin, immune booster, stomachache, TB, wounds	EVM
Aloe marlothii A. Berger; AM 1514	Asphodelaceae	Imvomvo	Tree	W	2.9	EVM	Leaf gel		
^a^ *Amaranthus hybridus* L.; AM 1515	Amaranthaceae	Nomdlomboyi	Herb	W	4.3	F	Leaves		
Artemisia afra Jacq. ex Willd.; AM 1516	Asteraceae	Umhlonyane	Shrub	W	24.6	M	Leaves, roots	Cough, diabetes, loss of appetite, TB	
*Asparagus africanus* L.; AM 1495	Asparagaceae	Umathunga	Climber	W	13.8	M	Leaves, roots	Sexually Transmitted Infections (STIs), to speed up labour, wounds	
*Asparagus asparagoides* (L.) Druce; AM 1433	Asparagaceae	Imvane	Climber	W	1.4	M	Roots	STIs	
Asparagus laricinus Burch.	Asparagaceae	Inqatha	Shrub	W	1.4	EVM	Roots		
^a^ *Beta vulgaris* L.; AM 1489	Chenopodiaceae		Herb	C	10.9	F	Leaves		
^a^ *Bidens pilosa* L.; AM 1536	Asteraceae	Umhlabangulo	Herb	W	3.6	F	Leaves	TB	M
Boophone disticha (L. f.) Herb.; AM 1496	Amaryllidaceae	Ishwadi	Herb	W	13.0	M	Bulb	Boils, circumcision wounds	EVM
Bowiea volubilis Harv. ex Hook. f. ssp. volubilis; AM 1490	Hyacinthaceae	Umagaqana	Herb	W	13.0	M	Bulb	Headache, inflammations, impotence	
^a^ *Brassica oleracea* L.; AM 1535	Brassicaceae	Ikhaphetshu	Herb	C	63.8	F	Leaves		
^a^Brassica rapa L.; AM 1534	Brassicaceae		Herb	C	2.9	F	Leaves		
*Bruguiera gymnorrhiza* (L.) Lam.; AM 1533	Rhizophoraceae	Isiqungati	Tree	W	4.3	T	Stems		
Bulbine abyssinica A. Rich.; AM 1492	Asphodelaceae	Uyakayakana	Herb	W	5.1	M	Leaves, roots	Diarrhoea, menstrual pain	EVM
Bulbine frutescens (L.) Willd.; AM 1532	Asphodelaceae	Ibhucu	Herb	W	8.7	M	Leaf sap	Diabetes, ringworm, wounds	EVM
Bulbine latifolia (L. f.) Roem. & Schult.; AM 1531	Asphodelaceae	Incelwane	Herb	W	15.2	M	Roots	Diarrhoea, speed up labour	EVM
*Capparis tomentosa* Lam.; AM 1410	Capparaceae	Umpasimani	Tree	W	5.8	M	Roots	Pneumonia, snakebite, sore throat	
^a^ *Capsicum annuum* L.; AM 1447	Solanaceae	Itshilisi	Herb	C	34.8	F	Fruits	Fever	M
Carissa bispinosa (L.) Desf. ex Brenan; AM 1448	Apocynaceae	Beta-umtumzi	Shrub	W	4.3	F	Fruits		
*Carpobrotus edulis* (L.) L. Bolus; AM 1449	Mesembryanthemaceae	Igcukuma	Shrub	W	7.9	M	Leaves	Ringworm, sore throat, TB, wounds	
^a^ *Catharanthus roseus* (L.) G. Don; AM 1450	Apocynaceae		Herb	WC	9.4	O	Leaves, whole plant	Cancer, diabetes	M
Centella coriacea Nannf.; AM 1453	Apiaceae	Unongotyozana	Herb	W	10.1	F	Leaves	STIs, TB, wounds	M, EVM
^a^ *Chenopodium album* L.; AM 1454	Chenopodiaceae	Iphunga	Herb	W	10.9	F	Leaves		
Cheilanthes hirta Sw.; AM 1455	Pteridaceae	Ifense	Pteridophyte	W	1.4	M	Leaves	Wounds	
^a^ *Citrus limon* (L.) Burm. f.; AM 1530	Rutaceae	Lamuni	Tree	C	18.8	F	Fruits, leaves	Skin rash	M
^a^Citrus sinensis (L.) Osbeck; AM 1529	Rutaceae	Iorenji	Tree	C	20.3	F	Fruits		
*Clivia miniata* Regel; AM 1434	Amaryllidaceae	Umayime	Herb	W	4.3	M	Leaves	Stomach problems	
Combretum erythrophyllum (Burch.) Sond.; AM 1528	Combretaceae	Umdubu	Tree	W	2.2	B	Leaves		
Convolvulus sagittatus Thumb; AM 1497	Convolvulaceae	Uboqo	Herb	W	1.4	M	Roots	Headache	
^a^ *Cucurbita maxima* Duchesne; AM 1517	Cucurbitaceae	Ithanga	Climber	C	23.9	F	Fruits		
^a^ *Cucurbita moschata* Duchesne ex Poir.; AM 1498	Cucurbitaceae	Ithanga	Climber	C	29.7	F	Fruits		
Cussonia paniculata Eckl. & Zeyh.; AM 1518	Araliaceae	Umsenge	Tree	W	5.8	M	Bark, leaves	Immune booster, skin diseases	
Cussonia spicata Thunb.; AM 1488	Araliaceae	Umgezisa	Tree	W	7.2	M	Leaves	Immune booster, stomach problems	EVM
*Cymbopogon nardus* (L.) Rendle; AM 1426	Poaceae	Umqungu	Grass	W	1.4	T	Leaves		
^a^ *Cynodon dactylon* (L.) Pers.; AM 1509	Poaceae	Uqaqaqa	Grass	W	4.3	B	Leaves		
^a^Datura stramonium L.; AM 1510	Solanaceae	Uvumbangwe	Herb	W	6.5	M	Leaves	Asthma, boils, wounds	
^a^Daucas carota L.; AM 1487	Apiaceae	umnqathi	Herb	C	26.8	F	Roots		
*Diospyros lycioides* Desf.; AM 1427	Ebenaceae	Umbhongisa	Shrub	W	2.2	B	Leaves		
*Dovyalis caffra* (Hook. f. & Harv.) Hook. f.; AM 1486	Flacourtiaceae	Incagolo	Shrub	W	4.3	F	Fruits		
Elephantorrhiza elephantina (Burch.) Skeels; AM 1425	Fabaceae	Intolwane	Shrub	W	17.4	M	Rhizomes	High blood pressure, haemorrhoids, rashes, purify blood	EVM
^a^ *Eucalyptus camaldulensis* Dehnh.; AM 1485	Myrtaceae		Tree	WC	5.1	T	Stems, leaves	Cough, TB	Fi, M
^a^Eucalyptus grandis W. Hill ex Maiden; AM 1519	Myrtaceae		Tree	WC	2.9	T	Stems		Fi
Euphorbia ingens E. Mey. ex Boiss; AM 1520	Euphorbiaceae	Intsema	Shrub	W	4.3	M	Latex	Cancer, skin rash	
^a^ *Ficus carica* L.; AM 1484	Moraceae	ikwiwane	Tree	C	5.1	F	Fruits		
Gnidia capitata L. f.; AM 1527	Thymelaeaceae	Isidikili	Shrub	W	3.6	M	Roots	Ringworm, wounds	
*Grewia occidentalis* L.f.; AM 1526	Malvaceae	Umvilani	Shrub	W	1.4	EVM	Roots		
Gunnera perpensa L.; AM 1525	Gunneraceae	Iphuzi	Herb	W	18.1	M	Rhizomes	Cancer, constipation, induce or augment labour, inflammation, menstrual pain, wounds	EVM
Harpephyllum caffrum Bernh.; AM 1483	Anacardiaceae	Umgwenye	Tree	W	12.3	F	Bark, fruits	Rash, wounds	EVM, M
Helichrysum gymnocomum DC.; AM 1458	Asteraceae	Icholachola	Herb	W	5.8	M	Whole plant	Colds, cough	
Helichrysum nudifolium (L.) Less.; AM 1457	Asteraceae	Icholocholo	Herb	W	8.7	M	Leaves	Cough, diabetes, menstrual pain, wounds	
Helichrysum odoratissimum (L.) Sweet; AM 1456	Asteraceae	Iphepho	Shrub	W	12.3	M	Whole plant	Colds, diabetes, headache, wounds	
Hermannia depressa N. E. Br.; AM 1435	Sterculiaceae	Phate eangaka	Shrub	W	3.6	M	Leaves	Colds, cough	
*Hyparrhenia hirta* (L.) Stapf; AM 1482	Poaceae	Umngcele	Grass	W	4.3	T	Leaves		
Hypoxis argentea Harv. ex Baker; AM 1499	Hypoxidaceae	Ixalanxa	Herb	W	5.8	M	Bulb	Cancer, ringworm, TB	EVM
Hypoxis hemerocallidea Fisch. Mey. & Ave-Lall.; AM 1524	Hypoxidaceae	Inongwe	Herb	W	24.6	M	Bulb	Cancer, diabetes, high blood pressure, immune booster, pimples	
Ilex mitis (L.) Radlk.; AM 1480	Aquifoliaceae	Isidumo	Tree	W	8.0	M	Bark	Sore throat, stomach problems	
^a^ *Ipomoea batatas* (L.) Lam.; AM 1428	Convolvulaceae	Bhatata	Climber	C	21.0	F	Root		
^a^Lactuca sativa L.; AM 1471	Asteraceae	Ilethasi	Herb	C	13.8	F	Leaves		
*Leonotis leonurus* (L.) R. Br.; AM 1523	Lamiaceae	Imvovo	Shrub	W	8.7	M	Leaves	Colds, cough, diarrhoea, snakebite	EVM
Lippia javanica (Burm. f.) Spreng.; AM 1479	Verbanaceae	Inzinziniba	Shrub	W	11.6	M	Leaves, roots	Chicken pox, colds, cough, wounds	
Lobelia flaccida (C. Presl) A. DC.; AM 1522	Campanulaceae	Itshilizi	Herb	W	2.2	M	Leaves	Abdominal pain	
^a^ *Lycopersicon esculentum* Mill.; AM 1521	Solanaceae	tumata	Climber	C	22.4	F	Fruits		
^a^Malus domestica Borkh.; AM 1481	Rosaceae	Apile	Tree	C	10.1	F	Fruits		
*Malva parviflora* L.; AM 1482	Malvaceae	Ujongelana	Shrub	W	1.4	M	Leaves	Wounds	
*Mentha longifolia* (L.) Huds.; AM 1483	Lamiaceae	Inxina	Herb	W	5.1	M	Leaves	Wounds	
*Miscanthus capensis* (Nees) Andersson; AM 1478	Poaceae	Idobo	Grass	W	4.3	T	Leaves, roots	Wounds	M
^a^Musa X paradisiaca L.; AM1503	Musaceae		Tree	C	13.0	F	Fruits		
^a^Nicotiana glauca Graham; AM 1502	Solanaceae	Icubamfene	Shrub	W	5.1	M	Leaves	Headache	
^a^ *Opuntia ficus-indica* (L.) Mill.; AM 1501	Cactaceae	Itolofiya	Tree	WC	24.6	F	Fruits, leaves, whole plant	Wounds	M, O
Pentanisia prunelloides (Klotzsch ex Eckl. & Zeyh.) Walp. ssp. prunelloides; AM 1411	Rubiaceae	Irubuxa	Herb	W	4.3	M	Roots	Wounds	
^a^ *Persea americana* Mill.; AM 1459	Lauraceae		Tree	C	11.6	F	Fruits, seed paste	Ringworm	M
^a^ *Phaseolus vulgaris* L.; AM 1460	Fabaceae	mbotyi	Herb	C	19.6	F	Fruits		
*Phoenix reclinata* Jacq.; AM 1469	Arecaceae	Isundu	Tree	W	3.6	C	Leaves, whole plant		O
*Phragmites australis* (Cav.) Steud.; AM 1500	Poaceae	Ingcongolo	Grass	W	4.3	T	Leaves		
^a^Physalis angulata L.; AM 1470	Solanaceae	Iyoli	Herb	W	5.1	M	Leaves	Burns	
^a^Pisum sativum L.; AM 1461	Fabaceae	Erityisi	Herb	C	15.9	F	Fruits		
*Pittosporum viridiflorum* Sims.; AM 1420	Pittosporaceae	Umkhwenkwe	Shrub	W	12.3	M	Bark, roots	Abdominal pain, cancer, fever	EVM
Plectranthus ambiguus (Bolus) Codd; AM 1429	Lamiaceae	Irhajojo	Herb	W	5.8	M	Leaves	Colds, cough	EVM
Podocarpus latifolius (Thunb.) R. Br. ex Mirb.; AM 1511	Podocarpaceae	Umcheya	Tree	W	1.4	EVM	Leaves		
*Prunus africana* Hook.; AM 1467	Rosaceae	Umkhakhazi	Tree	W	4.3	M	Bark, leaves	Cough, eye problems, TB	
^a^Prunus armeniaca L.; AM 1415	Rosaceae		Tree	C	12.3	F	Fruits		
^a^ *Prunus persica* (L.) Batsch; AM 1512	Rosaceae	Ipesika	Tree	C	13.0	F	Fruits, leaves	Eye problems	M
^a^ *Psidium guajava* L.; AM 1513	Myrtaceae	Ugwava	Shrub	WC	18.1	F	Fruits, leaves	Diarrhoea	M
Rhoicissus digitata (L.f.) Gilg & Brandt; AM 1468	Vitaceae	Uchithibhunga	Climber	W	7.2	M	Roots	Headache, high blood pressure	
^a^Ricinus communis L.; AM 1466	Euphorbiaceae	Umkakuva	Tree	W	5.1	M	Leaves	Stomachache	
Salvia scabra L. f.; AM 1416	Lamiaceae	Isicakathi	Herb	W	1.4	M	Leaves	Tonic	
Schotia latifolia Jacq.; AM 1418	Fabaceae	Umaphipha	Tree	W	4.3	T	Bark, stems	Diarrhoea	EVM, M
Senecio speciosus Willd.; AM 1419	Asteraceae	Idambiso	Herb	W	5.1	M	Leaves	Inflammations, wounds	
*Sida rhombifolia* L.; AM 1465	Malvaceae	Umdizawethafa	Shrub	W	1.4	M	Leaves	Wounds	
*Solanum aculeastrum* Dun.; AM 1417	Solanaceae	Umthuma	Shrub	W	5.1	M	Fruits	Boils, cancer, dysentery, impotence	
^a^Solanum nigrum L.; AM 1430	Solanaceae	Umsobo	Herb	W	6.5	M	Leaves	Ringworm, wounds	
^a^ *Solanum tuberosum* L.; AM 1444	Solanaceae	Amazambane	Herb	C	29.0	F	Tubers		
^a^ *Sonchus asper* (L.) Hill; AM 1445	Asteraceae	Irwabe	Herb	W	4.3	F	Leaves		
^a^Sonchus oleraceus L.; AM 1446	Asteraceae	Ihlaba	Herb	W	5.1	F	Leaves		
^a^ *Spinacia oleracea* L.; AM 1412	Amaranthaceae	Imifuno	Herb	C	54.3	F	Leaves		
*Sporobolus africanus* (Poir.) Robyns & Tournay; AM 1431	Poaceae	Umtshiki	Grass	W	3.6	T	Leaves		
*Sporobolus fimbriatus* (Trin.) Nees; AM 1436	Poaceae	Umgigwi	Grass	W	2.9	T	Leaves		
Syzygium cordatum Hochst. ex C. Krauss.; AM 1443	Myrtaceae	Umswi	Tree	W	5.8	M	Bark	Inflammation, pimples	
^a^ *Taraxacum officinale* Weber; AM 1463	Asteraceae	Ikhokhoyi	Herb	W	5.1	F	Leaves		
*Trichilia emetica* Vahl; AM 1437	Meliaceae	Umkhulu	Tree	W	3.6	M	Leaves	Wounds	
Tulbaghia acutiloba Harv.; AM 1462	Alliaceae	Isivumbampunzi	Herb	W	4.3	M	Leaves	Colic	
Tulbaghia alliacea L. f.; AM 1442	Alliaceae	Umwelela	Herb	W	5.8	M	Bulb	Boils, wounds	
Tulbaghia violacea Harv.; AM 1464	Alliaceae	Utswelane	Herb	W	7.2	M	Bulb	Cancer, TB	
Typha capensis (Rohrb.) N. E. Br.; AM 1438	Typhaceae	Ingcongolo	Herb	W	6.5	C	Leaves, rhizomes	Dysentery, STI	M
^a^Vitis vinifer L.; AM 1440	Vitaceae	Umdiliya	Climber	C	5.8	F	Fruits		
*Withania somnifera* (L.) Dunal; AM 1414	Solanaceae	Ubuvuma	Shrub	W	4.3	M	Leaves, roots	Inflammations, TB, wounds	
Xysmalobium undulatum (L.) W.T. Aiton; AM 1442	Apocynaceae	Itshongwe	Herb	W	5.1	M	Roots	STIs	
*Zantedeschia aethiopica* (L.) Spreng.; AM 1537	Araceae	Inyiba	Herb	W	3.6	M	Leaves	Wounds	
^a^ *Zea mays* L.; AM 1441	Poaceae	umbone	Grass	C	57.2	F	Seed		
Ziziphus mucronata Willd.; AM 1413	Rhamnaceae	Umphafa	Tree	W	10.9	M	Leaves	Chest pains, cough, dysentery, TB	EVM

^a^plant species that are exotic to South Africa

^b^Frequency (%) = percentage of participants mentioning plant species as useful; B = browse, C = brooms and crafts, *EVM*, ethnoveterinary medicine, *F* food plants, *Fi* firewood, *L* live fence and ornamental, *M* medicinal plants, *T* construction timber and thatching

**Table 3:** Plant families with the largest number of species (with at least 3 species) in the Eastern Cape province

Family	Number of species	%
Asteraceae	10	8.0
Fabaceae	9	7.2
Solanaceae	9	7.2
Poaceae	8	6.4
Asphodelaceae	7	5.6
Rosaceae	5	4.0
Apiaceae	4	3.2
Apocynaceae	4	3.2
Asparagaceae	4	3.2
Lamiaceae	4	3.2
Myrtaceae	4	3.2
Araliaceae	3	2.4
Malvaceae	3	2.4

### Plant use categories

**Fig. 3:**
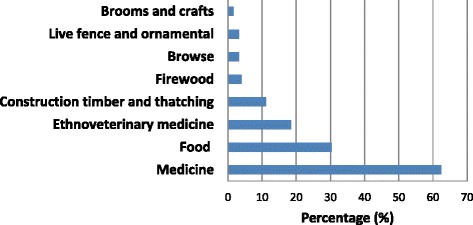
Use categories of plant species recorded in the Eastern Cape province

**Fig. 4:**
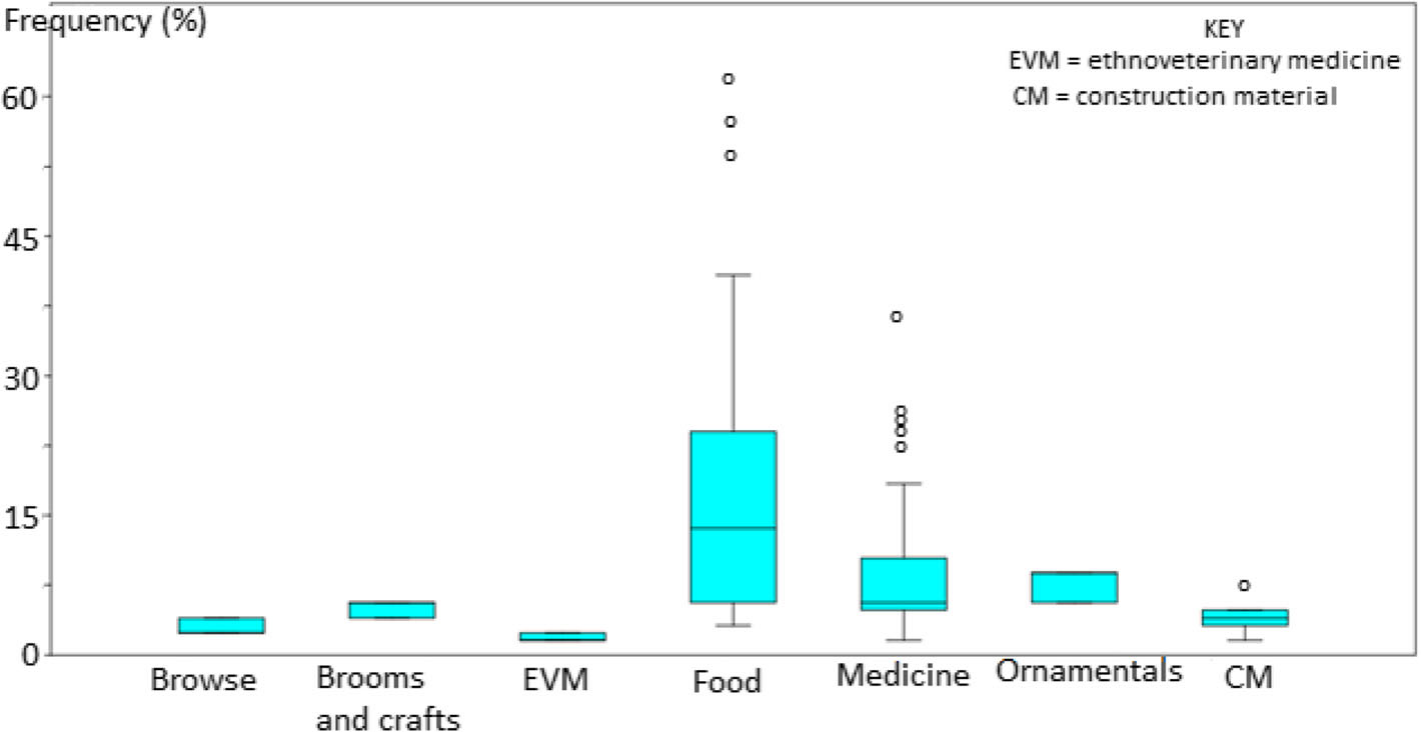
Percentage of species contributing to each of the main use categories

Most of the identified species were used as herbal medicines (62.4%), followed by food plants (30.4%), ethnoveterinary medicine (18.4%), construction timber and thatching (11.2%) (Fig. 3). PRA exercises with participants revealed that TEK about food plants in the Eastern Cape province was much higher than medicinal plants and other use categories (Fig. 4). These results corroborate previous research by Turreira-García et al. [32] which revealed that food plants are characterized by high direct-use values as they are used by households on a daily basis, diversity of food plants provide households with important sources of nutrients, help to reduce the need of buying marketed alternatives and play an important role in achieving household food security. The majority of recorded plant species (70.4%) in the Eastern Cape province had only one use, 23.2% had two uses and 6.4% had at least three uses. Multipurpose plant species mentioned by at least 15% of the participants included *Acacia karroo* browsed by livestock and wild animals, used as ethnoveterinary medicine, firewood and herbal medicine, *Aloe ferox* (ethnoveterinary medicine, herbal medicine), *Bulbine latifolia* (ethnoveterinary medicine, herbal medicine), *Capsicum annuum* (edible fruits, herbal medicine), *Citrus limon* (edible fruits, herbal medicine), *Elephantorrhiza elephantina* (ethnoveterinary medicine, herbal medicine), *Gunnera perpensa* (ethnoveterinary medicine, herbal medicine), *Opuntia ficus-indica* (edible fruits, herbal medicine, ornamental) and *Psidium guajava* (edible fruits, herbal medicine). These results corroborate earlier research findings which recognized *Acacia karroo* [33], *Aloe ferox* [34], *Bulbine latifolia* [35], *Capsicum annuum* [36], *Citrus limon* [37], *Elephantorrhiza elephantina* [38], *Gunnera perpensa* [39], *Opuntia ficus-indica* [40] and *Psidium guajava* [41] as multipurpose plant species.

### Medicinal plants

Plants used as ethnoveterinary and herbal medicines constituted 18.4% (23 species) and 62.4% (78 species) respectively of the total number of species recorded in this study. Plants used as ethnoveterinary and herbal medicines are culturally important plant use categories in the Eastern Cape province. The Xhosa people which constitute more than 80% of the population in the Eastern Cape province [21], is a cultural group known to use medicinal plants for cultural and religious practices [35, 42–46]. Previous research by Masika et al. [42] estimated that 75% of resource limited livestock farmers in the Eastern Cape province use traditional medicines to treat their animals and these farmers have a long history of treating and managing livestock diseases and ailments using ethnoveterinary medicines [43]. Masika and Afolayan [45] argued that this ethnoveterinary practice is an integral part of the Xhosa culture, a position that is unlikely to change to any significant degree in the coming years. About a third of species used as herbal medicines recorded in this study (30.8%) are highly valued medicinal plants in South Africa, their plant parts have potential in the development of new medicinal products with commercial value [47, 48], see Table 4. Therefore, some of plants used as herbal medicines in the Eastern Cape province have potential in the development of pharmaceutical products and drugs according to van Wyk [47] and van Wyk et al. [48]. Future research should attempt to correlate some of the documented ethnomedicinal uses of the species to their phytochemistry and pharmacological properties.

**Table 4:** Medicinal plants documented in this study that are considered to have potential in the development of new medicinal products with commercial value [47, 48]

Species	Plant parts used
*Acacia karroo*	Bark, exudate, leaf
*Alepidea amatymbica*	Root
*Aloe arborescens*	Leaf gel
*Aloe ferox*	Exudate, leaf gel
*Aloe marlothii*	Exudate
*Artemisia afra*	Essential oil, leaves
*Bulbine frutescens*	Leaf gel
*Carpobrotus edulis*	Leaves, fruits
*Elephantorrhiza elephantina*	Roots
*Gunnera perpensa*	Roots
*Helichrysum nudifolium*	Leaves
*Helichrysum odoratissimum*	Essential oil, leaves
*Hypoxis hemerocallidea*	Roots
*Leonotis leonurus*	Leaves
*Lippia javanica*	Leaves
*Mentha longifolia*	Leaves
*Pittosporum viridiflorum*	Bark
*Prunus africana*	Bark
*Tulbaghia alliacea*	Leaves
*Tulbaghia violacea*	Leaves
*Typha capensis*	Roots
*Withania somnifera*	Leaves, roots
*Xysmalobium undulatum*	Roots
*Ziziphus mucronata*	Leaves, roots

**Fig. 5:**
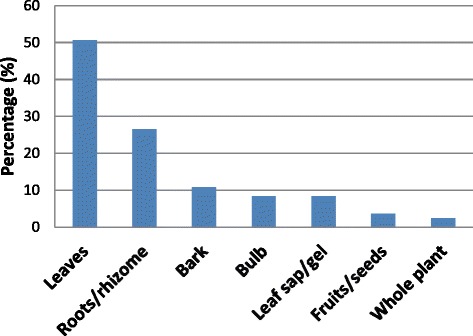
Plant parts used as ethnoveterinary and herbal medicine in the Eastern Cape province

The plant parts used for preparing ethnoveterinary and herbal medicines were the bark, bulbs, fruits, leaf gel, leaves, rhizomes, roots, sap, seeds and whole plant (Table 2). The leaves were the most frequently used plant parts (50.6%), followed by roots and rhizomes (26.5%), bark (10.8%), bulb and leaf gel and sap (8.4% each), fruits and seed (3.6%) and whole plant (2.4%) (Fig. 5). However, harvesting of bark, bulbs, rhizomes, roots and whole plants is not sustainable, particularly if such harvested plants are herbaceous. Such harvesting methods threaten the survival of the same plants used to treat human and animal diseases and ailments in the Eastern Cape province. It is well recognized by conservationists that medicinal plants primarily valued for their root parts and those which are intensively harvested for their bark, bulbs, rhizomes, roots or whole plants uprooted often tend to be the most threatened by over-exploitation [49]. Eight species widely used as ethnoveterinary and herbal medicines (9.6%) in the Eastern Cape province are threatened with extinction mainly because they are over-exploited for traditional medicine trade [44, 50]. These species include *Alepidea amatymbica*, *Boophone disticha*, *Bowiea volubilis* ssp. *volubilis, Clivia miniata*, *Gunnera perpensa*, *Hypoxis hemerocallidea*, *Ilex mitis* and *Prunus africana* [50]. The IUCN Red List Categories and Criteria version 3.1 of threatened species (http://www.iucnredlist.org) was used by Raimondo et al. [50] to assess the conservation status of these eight species, categorizing *Boophone disticha, Gunnera perpensa*, *Hypoxis hemerocallidea* and *Ilex mitis* as declining, *Alepidea amatymbica* (Endangered, A2d), *Bowiea volubilis* ssp. *volubilis* (Vulnerable, VUA2ad), *Clivia miniata* (Vulnerable, A2abcd) and *Prunus africana* (Vulnerable, A4acd, C1 + 2ai). According to Victor and Keith [51] and von Staden et al. [52], a species categorized as Least Concern (LC) under the IUCN Red List Categories and Criteria version 3.1 can additionally be flagged as of conservation concern either as rare, critically rare or declining, hence *Boophone disticha, Gunnera perpensa*, *Hypoxis hemerocallidea* and *Ilex mitis* were categorized as declining by Raimondo et al. [50]. All these eight species which are threatened are also harvested for the medicinal plant trade in the Eastern Cape province [44]. Other species used as ethnoveterinary and herbal medicines that are traded in the Eastern Cape province herbal medicine (muthi) markets include: *Asparagus africanus*, *A. asparagoides*, *Bulbine abyssinica*, *B. latifolia*, *Elephantorrhiza elephantina, Helichrysum odoratissimum*, *Pentanisia prunelloides* ssp. *prunelloides, Rhoicissus digitata*, *Tulbaghia alliacea* and *Xysmalobium undulatum* [44].

**Fig. 6:**
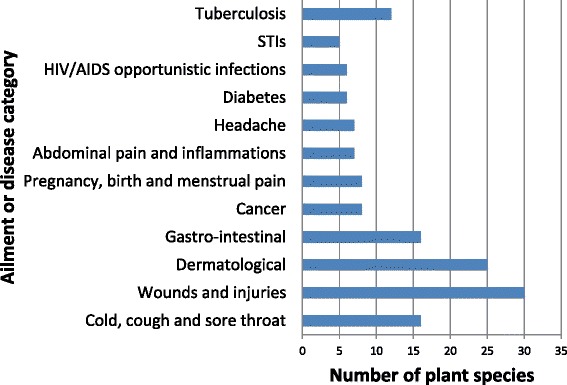
Major ailments or disease categories and plant species used. Some plant species were reported in more than one ailment or disease category

The majority of the plant species used as herbal medicines (39.7%) had a single therapeutic use, with 20 species (25.6%) used in the treatment of two diseases, 11 species (14.1%) treating three diseases, ten species (12.8%) treating four diseases and four species (5.1%) treating at least five diseases or ailments (Table 2). A total of 38 medical conditions were treated using remedies made from medicinal plants documented in this study. Wounds and injuries, dermatological, cold, cough, sore throat and gastro-intestinal problems, tuberculosis (TB), cancer, pregnancy, birth and menstrual pain, abdominal pain and inflammations, headache, HIV and AIDS opportunistic infections, diabetes and sexually transmitted infections (STIs) were treated with the highest number of medicinal plant species (Fig. 6). High usage of traditional medicines against TB and HIV and AIDS opportunistic infections is not surprising as these are leading causes of death in the Eastern Cape province [53]. Recent research by Statistics South Africa [54] also revealed that TB, diabetes mellitus and HIV and AIDS opportunistic infections are among the top ten leading causes of death in South Africa. The use of herbal and alternative medicine is increasing in South Africa, it is estimated that about 27% of the population use herbal remidies [55]. Therefore, this inventory of herbal medicines used in the Eastern Cape is a crucial starting point in trying to identify widely used herbal medicines for primary health care in the management of human and animal diseases. Such plant species are important candidates for further research aimed at developing effective drugs and pharmaceutical products for the treatment and management of human and animal diseases and ailments. One of the possible approaches to finding novel therapeutic agents is the screening of medicinal plants that are widely used in local communities to treat and manage human and animal diseases and ailments.

### Food plants

A variety of food plants were recorded in the Eastern Cape province, mainly edible fruits (21 species), leafy vegetables (10 species) and edible bulbs, roots or tubers (seven species) (Table 2). The majority of food plants documented in this study (71.1%) are exotic to South Africa (Table 2), widely cultivated in home gardens and agricultural fields in the Eastern Cape province as food plants. Exotic species which were collected from the wild included *Amaranthus hybridus*, *Bidens pilosa*, *Chenopodium album*, *Sonchus asper*, *S. oleraceus* and *Taraxacum officinale* (Table 2). Previous research by Jansen van Rensburg et al. [56, 57] and van der Hoeven et al. [58] showed that these five weedy species are widely collected as leafy vegetables in the wild in South Africa. With the exception of *Centella coriacea*, majority of indigenous food plants recorded in this study were fruit trees, which included *Carissa bispinosa*, *Dovyalis caffra* and *Harpephyllum caffrum* (Table 2). Some food plants such as *Allium sativum*, *Bidens pilosa, Capsicum annuum*, *Centella coriacea, Citrus limon*, *Harpephyllum caffrum*, *Opuntia ficus-indica, Persea americana*, *Prunus persica* and *Psidium guajava* were also used as herbal medicines. Important food plants mentioned by more than 20% of the participants included *Allium cepa* (onion), *Brassica oleracea* (cabbage), *Capsicum annuum* (pepper), *Cucurbita maxima* (pumpkin), *Cucurbita moschata* (butter-nut), *Daucas carota* (carrot), *Ipomoea batatas* (sweet potato), *Lycopersicon esculentum* (tomato), *Opuntia ficus-indica* (prickly pear), *Phaseolus vulgaris* (bean), *Solanum tuberosum* (potato), *Spinacia oleracea* (spinach) and *Zea mays* (maize). High frequencies associated with food plants in comparison with other plant use categories such as ethnoveterinary and herbal medicines (Fig. 4) corroborate research findings by Avila et al. [59] that the main purposes of agricultural environments of traditional communities is to produce food and the high agrobiodiversity found in these areas increases the nutritional diversity and quality of family diets.

### Other plant use categories

Interviews with participants in the Eastern Cape province revealed that *Acacia baileyana*, *A. caffra*, *A. dealbata*, *A. mearnsii*, *Bruguiera gymnorrhiza*, *Eucalyptus camaldulensis*, *E. grandis* and *Schotia latifolia* were used to construct huts, fence and different types of enclosures (Table 2). Grass species which included *Cymbopogon nardus*, *Hyparrhenia hirta*, *Miscanthus capensis*, *Phragmites australis*, *Sporobolus africanus* and *Sporobolus fimbriatus* were harvested to thatch traditional structures such as huts and enclosures (Table 2). Five species, namely *A. dealbata*, *A. karroo, A. mearnsii*, *Eucalyptus camaldulensis* and *E. grandis* were used as fuelwood and for space heating. Previous research by Chirwa et al. [60] on bioenergy use in the Eastern Cape province revealed that despite the high level of electrification in the province, firewood is still used in most households for cooking while electricity was mostly used for lighting. These authors argued that firewood is preferred for cooking food that takes a long time to prepare, while more convenient sources of energy such as electricity is used for short periods of cooking and re-heating of food. Similarly, Dold and Cocks [44] argued that fuelwood is still preferred in the Eastern Cape province for cooking traditional leafy vegetables because of the particular flavour it adds. Research by Dold and Cocks [35] revealed that food prepared for religious rituals must always be cooked with fuelwood, notably *Acacia karroo*. *Agave americana*, *Catharanthus roseus, Opuntia ficus-indica* and *Phoenix reclinata* were cultivated as live fence and ornamental plants by some households (Table 2). *Phoenix reclinata* and *Typha capensis* were used for making crafts such as mats and baskets. *Phoenix reclinata* leaves were shredded and bound together to make brooms. Leaves of *Acacia karroo*, *Combretum erythrophyllum*, *Cynodon dactylon* and *Diospyros lycioides* were browsed by livestock, mainly cattle and goats.

## Conclusion

PRA exercises with participants and observations made on main livelihood attributes in the study area seem to suggest that the TEK, practices and beliefs of the Xhosa people are dynamic and adaptive. This can be seen in the incorporation of exotic plant species to South Africa in the indigenous pharmacopoeia and indigenous diet of local people in the Eastern Cape province. Some exotic plants which are now part of the indigenous pharmacopoeia in the study area include *Agave americana*, *Catharanthus roseus, Ficus carica, Opuntia ficus-indica, Psidium guajava* and *Sonchus asper.* Research by Palmer [61] argued that the medicinal plant composition of a community is the product of experimentations conducted throughout the history of a community and represents an adaptation of this culture over time. While Alencar et al. [62] argued that any indigenous medical system is not a static social institution that is not evolving, as there is evidence of insertions and deletions of plants that compose it, with the addition of exotic plants as herbal medicines. Therefore, results of the current study corroborate an earlier observation that TEK systems are a reservoir of experiential knowledge that can provide important insights for the design of adaptation and mitigation strategies to cope with global environmental change [63].
